# The impact of Renin-Angiotensin System Inhibitors on bone fracture risk: a nationwide nested case-control study

**DOI:** 10.1186/s12891-023-07102-5

**Published:** 2024-01-02

**Authors:** Kwang Min Kim, Eun Jung Hwang, Sangjin Lee, Jeong-Hyun Yoon

**Affiliations:** 1 Department of Medicine, Samsung Changwon Hospital, Sungkyunkwan University School of Medicine, Changwon, South Korea; 2 College of Pharmacy and Research Institute for Drug Development, Pusan National University, 2, Busandaehak-Ro, 63 Beon-Gil, Geumjeong-Gu, 46241 Busan, South Korea; 3 Department of Statistics, College of Natural Science, Pusan National University, Busan, South Korea

**Keywords:** Renin-angiotensin system inhibitors, Angiotensin II receptor blockers, Fracture, Defined daily doses, Nested case–control study

## Abstract

**Background:**

The therapeutic efficacy of renin-angiotensin system inhibitors (RASi) in elderly patients with hypertension and at risk of fractures has been in the limelight because of accumulating evidence that localized RAS activation in bone tissue leads to osteoclastic bone resorption, resulting in osteoporosis. This study set out to investigate the association between RASi use and fracture incidence in a large cohort.

**Methods:**

We employed a nested case–control design to investigate the association between RASi use and newly developed fractures. A case was defined as a patient newly diagnosed with a fracture between January 2004 and December 2015. We selected 1,049 cases and controls using 1:1 propensity score matching. Conditional logistic regression analysis was conducted to estimate the association between RASi exposure and fracture incidence.

**Results:**

Overall, RASi usage was significantly associated with lower odds for fracture incidence (ever-users vs never-users: OR, 0.73; 95% CI, 0.59–0.91). We found that ARB-only users experienced fewer fractures than RASi-never users (OR, 0.65; 95% CI, 0.49–0.86), whereas ACEi-only users or ARB/ACEi-ever users did not. In subgroup analysis, RASi-ever users without cerebrovascular disease, those with a BMI exceeding 23, and statin exposure had significantly lower ORs.

**Conclusions:**

The present study established a significant association between RASi use and reduced fracture incidence, thus highlighting the potential clinical utility of RASi use as a preventive strategy in elderly patients at risk for osteoporotic fractures.

## Background

The global elderly population is growing at an unprecedented rate. Life expectancy worldwide is predicted to reach 76.2 years by 2050, a notable increase from 68.6 years in 2015 [1]. This growth presents numerous opportunities, but also poses significant public health challenges, including the prevalence of age-related chronic conditions like bone fractures. Serious fractures often lead to complications such as disability, pain, diminished quality of life, and even mortality [2]. Moreover, these fractures frequently necessitate hospitalization, imposing a significant financial burden on healthcare services and the broader economy. Thus, fracture prevention is crucial for the health and well-being of not only patients but also their caregivers. Bone fragility, largely associated with osteoporosis, often predisposes individuals to fractures. Osteoporosis, characterized by low bone mineral density (BMD) and deterioration of bone tissue microarchitecture, is a significant concern for both elderly males and females [3]. Its multifactorial pathogenesis involves genetics, aging, lifestyle, and environmental factors, with hypertension recognized as a major contributor [4, 5]. High blood pressure has been linked with abnormal calcium metabolism, potentially leading to increased calcium loss, secondary activation of parathyroid hormone, and consequent bone resorption [6, 7]. Given the potential link between hypertension and osteoporotic fractures, antihypertensive medications are garnering attention as potential therapeutic options for reducing fracture incidence. Notably, renin angiotensin system (RAS) inhibitors (RASi) such as angiotensin converting enzyme inhibitors (ACEi) and angiotensin II receptor blockers (ARBs) have shown promise in preclinical studies due to evidence of RAS activation influencing bone resorption and osteoporosis [8]. However, the capacity of RASi to protect against fractures in humans remains uncertain. Consequently, this study aims to investigate the associations between RASi use and fracture incidence in a large nationwide cohort.

## Methods

### Data source

Data analyzed in this study were obtained from the National Health Insurance Service-National Sample Cohort (NHIS-NSC) of South Korea, which was updated to version 2.0 in 2017. This large administrative cohort was created from the National Health Insurance (NHI) Database and has been described in detail previously [9, 10]. Briefly, NHIS-NSC is a nationwide, representative 2%, stratified, random sample of total NHI members that was created in 2006, comprising 1,025,000 subjects. Fourteen years (2002–2015) of information about health care utilization, including demographic information, diagnostic codes, procedures, and drug prescriptions is available for this cohort. Diagnoses were coded based on the International Statistical Classification of Disease and Related Health Problems, 10th revision (ICD-10). Furthermore, data on health behavior and medical history; anthropometric measurements such as height, weight, and BP; and blood test results for insured people are available for a subset of this cohort who underwent National Health Screening (NHS). The NHS service provides universal health care to approximately 97% of the Korean population. The participation rate of the eligible population in the NHIS health screening program was 74.8% in 2014 [11]. The use of selective and anonymized data for the present study was approved by the NHIS committee (NHIS-2021–2-110). This study protocol was approved by the Ethics Committee of Pusan National University and the study was conducted in accordance with the principles of the Declaration of Helsinki. As the study is based on retrospective analyses of existing anonymous administrative and clinical data, the requirement for informed subject consent was waived by the Institutional Review Board of Pusan National University (PNU IRB/2020_123_HR).

### Study design and population

**Fig. 1:**
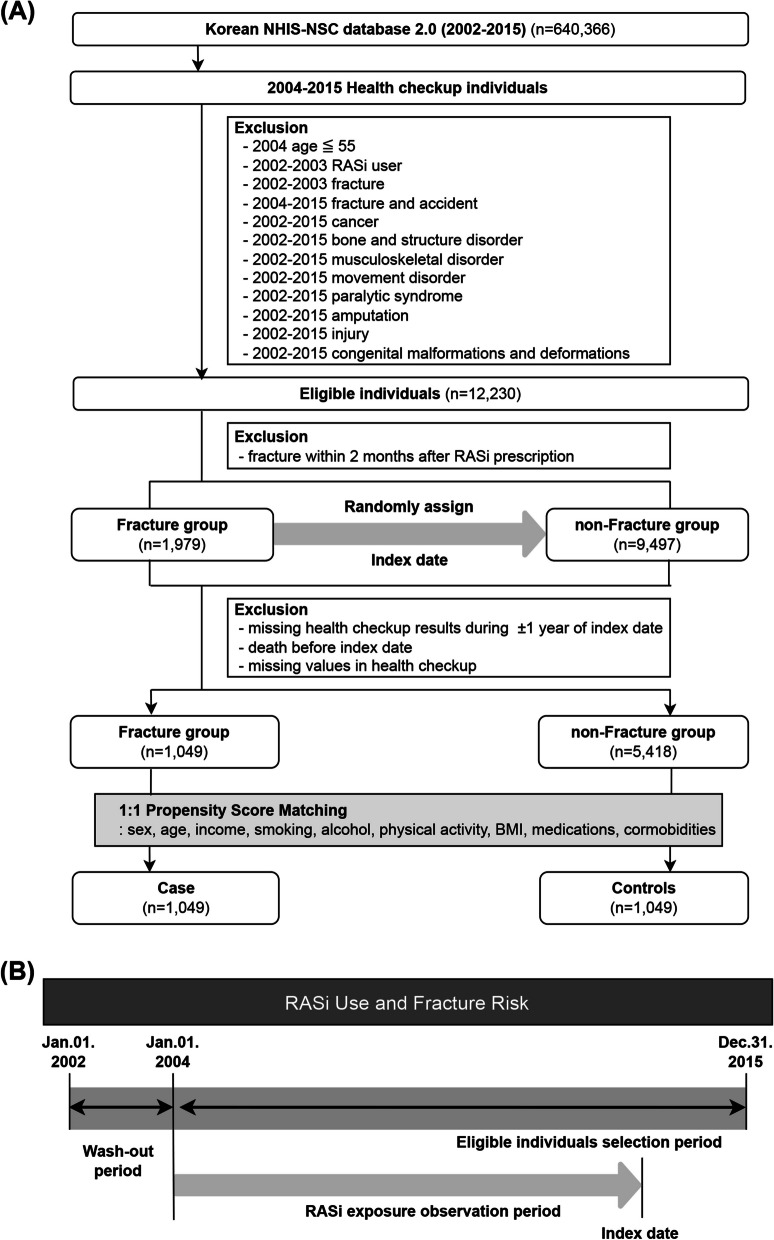
Diagram representing the nested case–control study design. **A** Flow chart of inclusion and exclusion criteria. **B** Schematic timeline of the study design

We employed a nested case–control design to investigate the association between RASi use and newly developed fractures. Out of one million overall subjects in the NHS-NSC 2.0 database, we selected 640,366 individuals who had undergone a national health checkup from January 2002 to December 2015 (the end point of this NHIS-NSC data set). Subsequently, we deliberately enrolled new RASi users and new-onset fracture cases by excluding subjects with a record of RASi prescriptions or fracture diagnosis in the 2 years prior to 2004 (implementing a washout period of 2 years, 2002–2003). We applied additional exclusion criteria as follows: ≤ 55 years of age as of 2004, fracture due to a traumatic accident even after the washout period, diagnosis of any underlying diseases, conditions, or congenital etiologies that could weaken bone tissue, possibly resulting in a fracture, and history of amputation or injury from 2002 to 2015. A total of 12,230 individuals were eligible and further exclusion was done based on a fracture diagnosis within 2 months after the first RASi prescription, which would have made determination of the influence of RASi difficult. Subsequently, a nested cohort was set up. We identified 1,979 study participants newly diagnosed with a fracture between January 2004 and December 2015 as our cases. The cohort index date of the case group was defined as the date when the fracture was first diagnosed. Non-fracture controls (*n* = 9,497) were randomly assigned a pseudo-index date corresponding to the index date of the fracture cases. Among these cases and controls, we additionally excluded subjects who lacked health checkup records within 1 year before and after the index date, had missing data on important covariates, and those who died before the index date. Of the 5,418 subjects who met all criteria for the non-fracture group, we delimited non-fracture cases using 1:1 propensity score matching (PSM). Based on national health checkup data collected within 1 year before and after the index date, one control subject with the same health checkup year, age, sex, income, smoking habits, alcohol consumption, physical activity, body mass index (BMI), use of medications, and presence of comorbidities was selected for each of the fracture cases. By matching health checkup years, the RASi exposure observation periods of the case and control groups were matched. Consequently, a total of 1,049 fracture cases and 1,049 matched controls were analyzed to determine the association between RASi use and fracture incidence (Fig. 1).

### Study outcomes and assessment of RASi use

The primary outcome of this study was the identification of fracture incidence among drug-naïve patients who started taking a RASi. To evaluate the association between RASi use and fracture incidence, we assessed exposure to RASi on the basis of prescriptions during the observation period from January 1, 2004 to the index date. A washout period of 2 years prior to 2004 (2002–2003) was set to eliminate any carryover effect of RASi use. RASi use was confirmed based on the prescription records in the NHIS-NSC; we included any RASi pharmaceutical product with a history of marketing in South Korea according to World Health Organization Collaborating Centre (WHOCC)-Anatomical Therapeutic Chemical (ATC) codes (https://www.whocc.no/atc_ddd_index/). Individuals were classified depending on their RASi exposure as ever-users (a daily dose for at least 30 or more days during the observation period) or never-users (lacked a prescription during the observation period). The risk of fracture was expressed as cases per population for RASi ever-user versus never-user groups. Secondary outcome measures were odds ratios (ORs) for fracture according to cumulative exposed RASi amount, cumulative duration, and average prescribed dose. These were calculated to identify the associations between the intensity of RASi dosage and fracture incidence. To investigate RASi effects related to dose, we estimated the overall amount of RASi exposure using the cumulative defined daily dose (cDDD), which was calculated as the sum of the defined daily dose (DDD) for all prescribed days. Additionally, cumulative prescription days were obtained to identify the exposure period to RASi. Furthermore, to investigate the usual daily amount of RASi exposure, we calculated the mean prescribed daily dose (PDD) as the average daily RASi dose dispensed to the subject regardless of RASi exposure period. Definitions of DDD and PDD were based on WHO criteria [12].

### Measurements and parameter definitions

Characteristics of cases and controls were based on health checkup results with matched dates. NHIS-NSC database includes a self-administered questionnaire covering smoking, alcohol consumption, and physical activity. The presence of underlying diseases known to affect the risk of osteoporotic fracture, including cardiovascular disease (CVD), cerebrovascular disease, chronic obstructive pulmonary disease (COPD), diabetes mellitus (DM), rheumatoid arthritis (RA), hyperthyroidism, chronic liver disease, malnutrition, and Crohn’s disease, were established when the ICD code of each disease was identified more than twice prior to the index date. Subjects were classified as non-drinkers or drinkers (never/ever) according to their reported alcohol consumption. Subjects were categorized into one of three groups according to current smoking status (none/past/current). Regular exercise was defined as ≥ 150 min of moderate intensity activity per week or ≥ 75 min or more of vigorous intensity activity per week. Some individual level covariates were converted to categorical variables to identify non-linear relationships with fractures as follows: age, body mass index (BMI), and income percentile. Participants were categorized into three age groups: younger-old (≤ 65 years), older (66–69 years), or oldest subjects (≥ 70 years). The four BMI groups according to the Asian-Pacific cutoffs were underweight (≤ 8.5 kg/m^2^), normal (18.5–22.9 kg/m^2^), overweight (23–24.9 kg/m^2^), or obese (≥ 25.0 kg/m^2^) with BMI calculated as weight/height^2^ [13]. The three household income groups were lower (1–3/10), middle (4–7/10), or upper (8–10/10). All other characteristics besides the health checkup results mentioned above were confirmed based on diagnosis and prescription records. Charlson comorbidity index (CCI) score, which includes a total of 17 comorbidities, was calculated based on ICD codes within 1 year prior to the index date [14]. An ever-user of hormone replacement therapy (HRT) was defined as possessing at least one prescription of any medication used for HRT before the index date; otherwise, the subject was classed as a never-user. This definition of drug usage was applied to all other types of drugs including calcium and vitamin D supplements, other anti-osteoporotic agents, glucocorticoids, thiazolidinediones, antithyroid drugs, antihypertensive agents, antiepileptics, selective serotonin reuptake inhibitors (SSRIs), tricyclic antidepressants (TCA), benzodiazepines, and statins.

### Statistical analysis

To minimize imbalances in the weighting of multiple confounders among subjects newly diagnosed with fracture and those with no fracture, case–control groups were matched by propensity scores. The standardized difference was used to quantify differences in means or prevalence rates between case and control groups. Matching was performed using nearest neighbor matching without replacement, with each individual diagnosed with a fracture matched to an individual without a fracture [15]. Baseline data, classified by fracture incidence into case and control groups, were summarized as frequencies (percentages) after PSM. Statistical differences in categorical variables between case and control groups were investigated by chi-square tests. Conditional logistic regression analysis was conducted to estimate the association between RASi exposure and fracture incidence, and ORs and 95% confidence intervals (CIs) were calculated. In subgroup analysis, baseline characteristics of cases and controls were compared and variables exhibiting significant differences were adjusted. A two-sided *P*-value of < 0.05 was considered statistically significant. Statistical analyses were carried out using SAS enterprise guide, version 7.1 (SAS Institute, Cary, North Carolina, USA) and R version 3.5 (Vienna, Austria; http://www.R-project.org/).

## Results

Table 1 presents the baseline characteristics of fracture cases and controls after PSM. Men outnumbered women in both fracture incidence cases and controls: 697 (66.4%) to 352 (33.6%) and 690 (65.8%) to 359 (34.2%), respectively. Roughly one third of the study population was 66–70 years old (36.6%) and had a normal BMI (40.9%). All matching variables, including sex, age, BMI, alcohol consumption, smoking habits, physical activity, income, comorbidities, underlying diseases, presence of malnutrition, and prescription drug use, were evenly distributed between fracture cases and controls. Statistical comparisons of these variables revealed no significant differences, confirming that our matching process effectively created comparable cohorts.

**Table 1:** Baseline characteristics of fracture cases and matched controls

Variables	Fracture case		Controls		*P*-value
	**(*n* = 1,049)**		**(*n* = 1,049)**		
	**n**	**%**	**n**	**%**	
**Age, years**					0.519
≤ 65	351	33.5	342	32.6	
66–70	391	37.3	376	35.8	
≥ 71	307	29.3	331	31.6	
**Sex**					0.782
Men	697	66.4	690	65.8	
Women	352	33.6	359	34.2	
**BMI, kg/m**^**2**^					0.991
< 18.5	34	3.2	35	3.3	
18.5–22.9	429	40.9	430	41.0	
23.0–24.9	277	26.4	271	25.8	
≥ 25.0	309	29.5	313	29.8	
**Alcohol consumption**					0.626
None	619	59.0	607	57.9	
Drinker	430	41.0	442	42.1	
**Smoking**					0.986
Non-smoker	604	57.6	607	57.9	
Past smoker	213	20.3	210	20.0	
Current smoker	232	22.1	232	22.1	
**Regular exercise**					0.403
No	335	31.9	354	33.7	
Yes	714	68.1	695	66.3	
**Household income class**					0.777
Lower	274	26.1	263	25.1	
Middle	358	34.1	372	35.5	
Upper	417	39.8	414	39.5	
**CCI score**					0.702
0	262	25.0	283	27.0	
1	233	22.2	221	21.1	
2	180	17.2	170	16.2	
≥ 3	374	35.7	375	35.7	
**Comorbidity**					
CVD	187	17.8	179	17.1	0.687
Cerebrovascular disease	125	11.9	120	11.4	0.786
COPD	90	8.6	105	10.0	0.292
Diabetes mellitus	350	33.4	342	32.6	0.745
Rheumatic arthritis	92	8.8	112	10.7	0.161
Hyperthyroidism	47	4.5	46	4.4	1.000
Chronic liver disease	457	43.6	451	43.0	0.826
Malnutrition	7	0.7	5	0.5	0.772
Crohn’s disease	265	25.3	268	25.5	0.920
**Medications other than RASi**					
Calcium/vitamin D supplements	76	7.2	80	7.6	0.803
Other osteoporotic agents	14	1.3	15	1.4	1.000
HRT agents	23	2.2	20	1.9	0.758
Glucocorticoids	68	6.5	62	5.9	0.651
Thiazolidinedione	34	3.2	27	2.6	0.436
Antithyroid drugs	10	1.0	8	0.8	0.813
Beta blockers	240	22.9	230	21.9	0.637
Calcium channel blockers	380	36.2	392	37.4	0.618
Loop diuretics	57	5.4	50	4.8	0.552
Thiazides	226	21.5	227	21.6	1.000
Antiepileptics	133	12.7	148	14.1	0.369
SSRIs	29	2.8	33	3.1	0.699
TCAs	145	13.8	150	14.3	0.802
Benzodiazepines	659	62.8	660	62.9	1.000
Statins	231	22.0	225	21.4	0.791

*Abbreviations*: *CCI* Charlson comorbidity index, *CVD* Cardiovascular disease, *COPD* Chronic obstructive pulmonary disease, *RASi* Renin-angiotensin system inhibitors, *HRT* Hormone replacement therapy, *SSRI* Selective serotonin reuptake inhibitor, *TCA* Tricyclic antidepressant

The association between fracture incidence and RASi usage was assessed using conditional logistic models (Table 2). The frequency of RASi-ever users in fracture cases and controls was 45.9% (*n* = 230/1,049) and 54.1% (*n* = 271/1,049), respectively. Overall, RASi usage was significantly associated with lower odds for fracture incidence (ever-users vs never-users: OR, 0.73; 95% CI, 0.59–0.91). RASi use significantly decreased the fracture incidence in current users, where RASi-ever users were divided into current and past users based on the presence of a RASi prescription on the index date. RASi use was also associated with a significant reduction in the risk of fracture in most case categories, whether classified according to cumulative dose, prescription period, or mean daily dose. However, certain specific RASi cumulative doses and cumulative prescription days intervals did not show a significant association with reduced risk of fracture (cDDD ≥ 365: OR, 0.94; 95% CI, 0.70–1.26; cumulative prescription days, ≥ 90 and < 180: OR 0.61; 95% CI, 0.37–1.03; cumulative prescription days, ≥ 365: OR, 0.95; 95% CI, 0.71–1.28). In a subsample of 1,387 men and 711 women analyzed based on sex, RASi use was not associated with a lower OR for fracture incidence. RASi use also did not differently affect fracture incidence among the three age groups (Table 3).

**Table 2:** Use of renin-angiotensin system inhibitors and incidence of fracture

Variables		Fracture case		Controls		Conditional logistic regression	
		**(*n* = 1,049)**		**(*n* = 1,049)**			
		**n**	**%**	**n**	**%**	**OR (95% CI)**	***P*-value**
RASi use	Never-user	819	51.3	778	48.7		
	Ever-user	230	45.9	271	54.1	0.73 (0.59–0.91)	0.004
RASi prescription at the index date	Never-user	819	51.3	778	48.7		
	Past user	67	46.5	77	53.5	0.72 (0.49–1.05)	0.084
	Current user	163	45.7	194	54.3	0.73 (0.57–0.94)	0.014
RASi cumulative dose (cDDD)	0 < cDDD < 90	44	40.0	66	60.0	0.65 (0.43–0.99)	0.043
	90 ≤ cDDD < 180	26	36.6	45	63.4	0.53 (0.31–0.90)	0.018
	180 ≤ cDDD < 365	28	37.3	47	62.7	0.47 (0.27–0.82)	0.007
	cDDD ≥ 365	132	53.9	113	46.1	0.94 (0.70–1.26)	0.693
Cumulative prescription days	0 < days < 90	43	37.7	71	62.3	0.60 (0.39–0.90)	0.014
	90 ≤ days < 180	30	41.7	42	58.3	0.61 (0.37–1.03)	0.063
	180 ≤ days < 365	27	35.5	49	64.5	0.49 (0.29–0.83)	0.009
	Days ≥ 365	130	54.4	109	45.6	0.95 (0.71–1.28)	0.744
Mean prescribed daily dose (PDD)	PDD < 0.5	7	46.7	8	53.3	0.84 (0.29–2.47)	0.752
	0.5 ≤ PDD < 1	167	44.5	208	55.5	0.72 (0.56–0.92)	0.008
	PDD ≥ 1	56	50.5	55	49.5	0.75 (0.49–1.15)	0.194

*Abbreviations*: *RASi* Renin-angiotensin system inhibitors, *cDDD* Cumulative defined daily dose, *PDD* Prescribed daily dose

**Table 3:** Use of renin-angiotensin system inhibitors and incidence of fracture by age and gender

Variables		Fracture case		Controls		Conditional logistic regression	
		**(*n* = 1,049)**		**(*n* = 1,049)**			
		**n**	**%**	**n**	**%**	**OR (95% CI)**	***P*-value**
Age (≤ 65 years)	Never-user	295	51.7	276	48.3		
	Ever-user	56	45.9	66	54.1	0.80 (0.42–1.51)	0.486
Age (66–70 years)	Never-user	304	51.9	282	48.1		
	Ever-user	87	48.1	94	51.9	0.51 (0.27–0.96)	0.036
Age (≥ 71 years)	Never-user	220	50.0	220	50.0		
	Ever-user	87	43.9	111	56.1	0.59 (0.31–1.12)	0.109
Male	Never-user	545	51.6	512	48.4		
	Ever-user	152	46.1	178	53.9	0.74 (0.55–1.00)	0.050
Female	Never-user	274	50.7	266	49.3		
	Ever-user	78	45.6	93	54.4	0.67 (0.41–1.10)	0.114

To evaluate the influence of RASi use on fracture incidence across different RASi types, we stratified fracture cases and controls according to use of ARBs-only, ACEi-only, or both agents (Table 4). We found that ARB-only users experienced fewer fractures than RASi-never users (OR, 0.65; 95% CI, 0.49–0.86). Contrarily, a significant association was not found between other RASi types and fracture incidence (ACEi-only users: OR, 0.85; 95% CI, 0.55–1.30; ARB- and ACEi-users: OR, 0.82; 95% CI, 0.54–1.26).

**Table 4:** Incidence of fracture according to type of renin-angiotensin system inhibitor

Variables		Fracture case		Controls		Conditional logistic regression	
		**(*n* = 1,049)**		**(*n* = 1,049)**			
		**n**	**%**	**n**	**%**	**OR (95% CI)**	***P*-value**
RASi use	Never-user	819	51.3	778	48.7		
	ARB only-user	126	44.1	160	55.9	0.65 (0.49–0.86)	0.003
	ACEi only-user	51	47.7	56	52.3	0.85 (0.55–1.30)	0.447
	ARB/ACEi-ever user	53	49.1	55	50.9	0.82 (0.54–1.26)	0.369

*Abbreviations*: *RASi* Renin-angiotensin system inhibitors, *ARB* Angiotensin II receptor blocker, *ACEi* As angiotensin converting enzyme inhibitor

Finally, we conducted subgroup analyses to determine if there were changes in the relationship between RASi use and fracture incidence in the presence of specific clinical conditions (Table 5). These subgroups included cases with the presence or absence of DM, CVD, exposure to statins, and BMI exceeding 23. ORs were significantly lower for RASi-ever users without CVD, those with a BMI exceeding 23, and those exposed to statins.

**Table 5:** Risk of fracture considering exposure to renin-angiotensin system inhibitors in subgroups

Variables		Fracture case		Controls		Conditional logistic regression	
		**(*n* = 1,049)**		**(*n* = 1,049)**			
		**n**	**%**	**n**	**%**	**OR (95% CI)**	***P*-value**
Diabetes	Never-user	215	52.7	193	47.3		
	Ever-user	135	47.5	149	52.5	0.81 (0.49–1.35)	0.415
No diabetes	Never-user	604	50.8	585	49.2		
	Ever-user	95	43.8	122	56.2	0.74 (0.51–1.07)	0.108
No stroke	Never-user	811	51.2	773	48.8		
	Ever-user	223	45.7	265	54.3	0.73 (0.58–0.91)	0.005
BMI ≥ 23 kg/m^2^	Never-user	427	51.9	396	48.1		
	Ever-user	159	45.8	188	54.2	0.65 (0.46–0.91)	0.013
Statins	Never-user	66	50.8	64	49.2		
	Ever-user	92	48.4	98	51.6	0.62 (0.47–0.83)	0.001
No statins	Never-user	392	50.6	382	49.4		
	Ever-user	71	46.1	83	53.9	0.61 (0.35–1.05)	0.074

*Abbreviations*: *BMI* Body mass index

## Discussion

This study was designed to evaluate the association between RASi use and fracture incidence via a large, population-based, nested case–control study. With the global population aging, the number of individuals suffering from chronic conditions is set to rise. Among these conditions, fractures are likely to pose a significant challenge for clinicians and emerge as a crucial health issue in the coming decades. Metabolic bone disorders primarily result from abnormalities in calcium and phosphorus metabolism, imbalance in parathyroid hormone (PTH), and deficiency in vitamin D, an important mediator of calcium metabolism [16]. Epidemiological evidence and research suggest a connection between hypertension and vitamin D deficiency, which could potentially accelerate the age-related decrease in bone density [17]. Consequently, thiazide diuretics or beta-blockers may decrease fracture incidence by reducing the risk of renal calcium excretion in elderly hypertensive individuals [18, 19]. RASis have also been proposed to lessen the risk of fractures due to their blood pressure controlling ability [20]. Experimental data shows that RAS inhibition improves bone quality independently of the effect of RAS inhibitors on blood pressure. Initially, the function of the RAS was assumed to be primarily systemic. However, recent studies have revealed that increased local activation of the RAS can lead to osteoporosis via osteoclast activation. The vascular systems have a fundamental role in bone remodeling, and thus, blood flow regulation is another osteoprotective effect of the RAS. RASis have garnered significant interest as these medications are already widely used, safe, and reasonably priced. Nevertheless, inconsistencies persist in the reported associations between RASi use and the risk of fractures. For instance, Kunutsor et al. reported that RASi use was not associated with a long-term risk of composite fracture and that these inhibitors had limited beneficial effects [21]. On the contrary, other studies have reported that RASis can decrease the risk of fractures in the elderly population [22, 23]. This study contributes to this debate by providing relevant information based on data analyses from a population-based cohort. Overall, we discovered a significant relationship between RASi exposure and a decreased risk of fracture incidence that remained consistent across multiple clinically relevant subgroups. Our data was gathered from a large, representative, nationwide registry, accurately reflecting real-world clinical situations with a relatively low attrition rate. This provided an unbiased evaluation of RASi exposure data prior to the incidence of fracture, and the use of registry data helped to eliminate the potential for recall bias. The current study's concept is a culmination of prior analyses; therefore, we opted for a nested case–control study model and utilized an adjusted regression model to minimize potential confounding effects. As a result, our study design was comprehensive and robust, and statistical power was preserved even during subgroup analyses. Our study acknowledges the role of cognitive impairments in increasing the risk of fractures, not solely due to falls but also through decreased mobility and overall frailty. However, the retrospective nature of our study and the specific dataset used (NHIS-NSC) limited our capacity to include detailed clinical data such as diagnoses of dementia or Alzheimer's disease. Despite these limitations, we believe our study provides a foundational understanding of the relationship between RASi use and fracture risk, and we highlight the need for future research to incorporate a broader range of cognitive risk factors.

Our analyses yielded several notable findings. Firstly, the incidence of fractures was diminished among current RASi users as of the index date. For past RASi users, we posited less RASi cumulative defined daily dose (cDDD) because the nature of the data set prevented us from precisely determining these subjects’ RASi exposure period. The findings of the present study, demonstrating a significant reduction in fracture risk even among those with a comparatively low cDDD, bolster this assumption. Angiotensin II exerts a stimulatory influence on osteoblasts and is thus suggested to have adverse effects on bone structure by enhancing bone resorption [24]. It also reduces the uptake of calcium into bones, impedes osteoblastic cell differentiation and bone formation, and diminishes alkaline phosphatase activity [25]. It appears plausible that the inhibitory effect of RASi on angiotensin II signal transduction could be dose-independent, potentially preventing osteoporosis, augmenting bone mass, and expediting bone healing. Secondly, notwithstanding the relationship we uncovered between RASi exposure and diminished fracture incidence, we did not observe a significant reduction in fracture risk with increased RASi cumulative doses (cDDD) or prescription durations exceeding 365 days, which stands in contrast to our initial expectations. Interpreting this unexpected result is challenging, especially since a significant association was not evident in the highest quartile, as guided by cumulative dosage and prescription intervals. We can speculate that patients receiving higher RASi doses over a prolonged period might be older and have been managing chronic diseases for a longer duration. This apparent contradiction could be attributed to the potential residual confounding effects of other factors, possibly undermining the anticipated inverse correlation between RASi use and fracture incidence. We propose that the beneficial effects of a medication may not always intensify linearly with its duration of use or dosage. Furthermore, the effects of a medication like RASi could intertwine with complex biological pathways, potentially influenced by an individual’s fluctuating health status, thus potentially altering the medication’s effectiveness and its role in fracture risk. For example, elderly patients prescribed higher RASi doses for longer durations could be more prone to prolonged bouts of chronic illness. Consequently, it’s essential to recognize the observational nature of our findings, which may not account for every potential confounding variable. While our analyses did not reveal a significant difference in the effect of RASi use on fracture incidence between men and women, we observed that the proportion of men in the fracture cases was higher than that of women, which contrasts with typical epidemiological trends of higher osteoporotic fracture incidence in women. This observation may be influenced by the stratified random sampling of the national population in the NHIS-NSC database, the inclusive nature of our fracture criteria, and the higher screening participation rate among men. It is important to note that while our propensity score matching was effective in controlling for various fracture risk factors, it was not specifically designed to address gender balance. This aspect is important for interpreting the study results, particularly when extrapolating to osteoporosis in a typically female-dominated population. Prolonged RASi users might grapple with more severe underlying health problems, possibly heightening their fracture risk and obscuring the benefits of RASi on fracture risk reduction we initially perceived. Thirdly, RASi use did not significantly reduce the risk of fracture incidence in men versus women. However, fracture risk tends to be lower in men than women. This outcome might be partially explained by sex differences in the RAS. Sex hormones affect components of the RAS via several mechanisms. The higher activity level of RAS in men compared to women is well-established [26, 27]. Therefore, we posit that our finding of significantly reduced fracture incidence in men using RASi is related to the higher RAS activity in men than women. Fourthly, we discovered that ARB-only use had a more considerable impact on reducing the risk of fracture incidence than ACEi-only use or ARB/ACEi-ever use. The reason for this is unclear; however, it could be because angiotensin II commonly induces the expression of the receptor activator of nuclear factor kappa B ligand (RANKL) in osteoblasts, leading to the activation of osteoclasts [28], and this process might be more effectively blocked by ARBs than ACEi. Our results align with those of Kwok et al., who found that ARB users displayed a lower fracture incidence than ACEi users among older hypertensive men [29].

While the current study established a link between RASi use and a reduction in fracture incidence, it possesses several limitations. Firstly, the NHIS-NSC database lacks comprehensive information about fractures, including cause, site, severity, and BMD data. The inclusion of these variables in future research could yield a more thorough understanding of the relationship between RASi use and fractures. This could also reveal differences in the effectiveness of RASi in preventing various types of fractures and illuminate the influence of RASi on bone health. Secondly, although this study offered significant insights into the relationship between RASi usage and fracture incidence, it didn’t consider factors such as medication adherence and renal function markers. Accounting for these in future investigations may provide a more complete understanding and potentially affect our comprehension of the RASi efficacy and its association with fracture risk. Thirdly, due to the frequent switching of ACEi among subjects, we could not assess fracture risk in ACEi-only users. Lastly, we did not evaluate the frequency of calcium or vitamin D3 supplement usage, which are available as over-the-counter medicines in South Korea and could potentially associate with fracture incidence.

## Conclusions

The present study established a significant association between RASi use and diminished fracture incidence in a nationwide, population-based, real-world setting. Our findings emphasize the clinical potential of RASi as a preventive strategy for osteoporotic fractures in elderly patients at risk. Future studies, balanced and comprehensive in nature, are required to verify the capacity of RASi to reduce fracture risk.

## Data Availability

This study was performed using the National Health Insurance System (NHIS) database (https://nhiss.nhis.or.kr/), and the results do not necessarily represent the opinion of the National Health Insurance Corporation. Restrictions apply to the availability of these data, which were used under license for this study. The datasets generated and/or analyzed during the current study are not publicly available as current study are from the Korean National Health Insurance which is not publicly available due to participant privacy concern but are available from the corresponding author on reasonable request.
